# Alloying Effects on Charge-Carrier Transport in Silver–Bismuth Double Perovskites

**DOI:** 10.1021/acs.jpclett.3c02750

**Published:** 2023-11-10

**Authors:** Marcello Righetto, Sebastián Caicedo-Dávila, Maximilian T. Sirtl, Vincent J.-Y. Lim, Jay B. Patel, David A. Egger, Thomas Bein, Laura M. Herz

**Affiliations:** † Department of Physics, Clarendon Laboratory, University of Oxford, Parks Road, Oxford OX1 3PU, United Kingdom; ‡ Physics Department, TUM School of Natural Sciences, Technical University of Munich, James-Franck-Straße 1, Garching 85748 Germany; § Department of Chemistry and Center for NanoScience (CeNS), University of Munich (LMU), Butenandtstr. 11, 81377 Munich, Germany; ∥ Institute for Advanced Study, Technical University of Munich, Lichtenbergstrasse 2a, D-85748 Garching, Germany

## Abstract

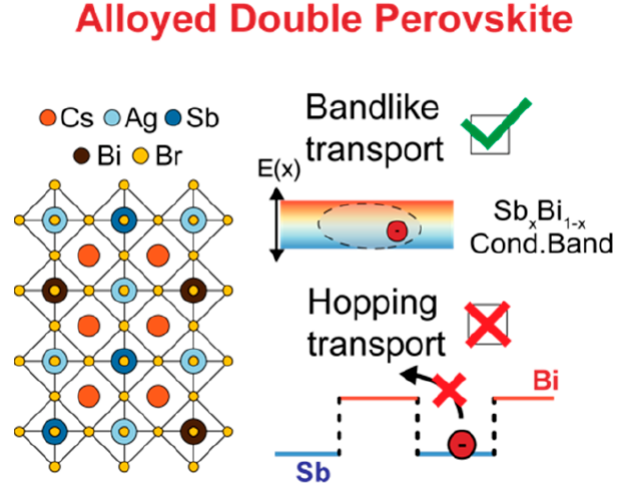

Alloying is widely adopted for tuning the properties of emergent semiconductors for optoelectronic and photovoltaic applications. So far, alloying strategies have primarily focused on engineering bandgaps rather than optimizing charge-carrier transport. Here, we demonstrate that alloying may severely limit charge-carrier transport in the presence of localized charge carriers (e.g., small polarons). By combining reflection–transmission and optical pump–terahertz probe spectroscopy with first-principles calculations, we investigate the interplay between alloying and charge-carrier localization in Cs_2_AgSb_*x*_Bi_1–*x*_Br_6_ double perovskite thin films. We show that the charge-carrier transport regime strongly determines the impact of alloying on the transport properties. While initially delocalized charge carriers probe electronic bands formed upon alloying, subsequently self-localized charge carriers probe the energetic landscape more locally, thus turning an alloy’s low-energy sites (e.g., Sb sites) into traps, which dramatically deteriorates transport properties. These findings highlight the inherent limitations of alloying strategies and provide design tools for newly emerging and highly efficient semiconductors.

Silver–bismuth halides have recently emerged as a promising new family of semiconductors with potential applications in photovoltaics,^1,2^ photocatalysis,^3,4^ and photodetectors.^5,6^ These materials have gained considerable attention owing to their nontoxic inorganic composition,^7,8^ low-temperature synthesis,^1,8^ and high stability,^7^ making them ideal alternatives to lead halide perovskites.^9^ However, the photovoltaic performance of silver–bismuth halides has been lagging behind with respect to their lead-based counterparts.^10^ For instance, record power-conversion efficiencies (PCE) just above 6% have been reported for silver–bismuth double perovskite Cs_2_AgBiBr_6_,^11−14^ trailing behind the 26.1% PCE reported for lead halide perovskites.^15^ Similarly, other silver–bismuth halides such as rudorffites (e.g., AgBiI_4_, Ag_3_BiI_6_, and Ag_2_BiI_5_)^16,17^ have reported PCEs below 6%.^18,19^

Following the well-established strategies developed for conventional semiconductors, alloying has been proposed as a promising approach to tune and improve the optoelectronic properties of several silver–bismuth halides and other double perovskites.^20−23^ The partial replacement of Ag^+^ and Bi^3+^ cations by other isovalent cations—such as Cu^+^, Na^+^, In^3+^, and Sb^3+^ —has proved effective in engineering bandgaps and tuning internal strains for this family of semiconductors.^20,24,25^ In 2020, Li et al. successfully replaced Bi^3+^ with Sb^3+^ cations in the Cs_2_AgBiBr_6_ double perovskite, yielding a bandgap lowering by up to 170 meV for improved solar spectral matching.^26^ Interestingly, the authors reported a bandgap bowing (i.e., narrower bandgap for intermediate alloy compositions) and ascribed it to the formation of type II band alignment between Sb and Bi sites. These observations confirmed initial reports for trivalent metal alloying in silver–bismuth double perovskites by Mitzi and co-workers.^20^ As explained by Cheetham and co-workers, by replacing Bi^3+^ 6p–Br^–^ 4p antibonding orbitals with higher-lying Sb^3+^ 5p–Br^–^ 4p ones yields a lower conduction band minimum at the *L* point.^8^ Furthermore, increased overlap between Sb^3+^ 5s and Br^–^ 4p orbitals owing to the reduced spin–orbit coupling (SOC) in Sb results in a higher-lying valence band maximum.^20,26^ On the other hand, the Sb^3+^ substitution in AgBi_2_I_7_ rudorffites yielded a considerable blue-shift of the bandgap (from ∼1.6 to ∼2 eV),^16^ thus revealing the strong dependence of this strategy on the orbital hybridization and the nature of optical transitions involved.

Despite recent successes of alloying strategies in bandgap engineering of silver–bismuth halides, only few studies have investigated the impact of alloying on charge-carrier transport properties.^27,28^ First-principles calculations for Bi/Sb alloying predicted improved phonon-limited mobilities with higher Sb concentrations, owing to the highly dispersive band associated with antimony.^8,26,28^ Similar trends were predicted for Bi/In alloying.^20,28^ Conversely, the only experimental study on charge-carrier transport in alloyed Bi/Sb double perovskites did not report significant charge-carrier mobility trends with changes in the Sb fraction, potentially owing to extrinsic (i.e., defects and morphology) effects.^27^ Investigating charge-carrier transport in alloyed silver–bismuth halides is even more critical in light of the strong coupling of charge carriers to the lattice exhibited by these materials.^29^ Some of us recently demonstrated that these strong charge-carrier phonon interactions profoundly affect charge-carrier transport in silver–bismuth double perovskites (Cs_2_AgBiBr_6_)^30^ and rudorffites (AgBiI_4_, Cu_2_AgBiI_6_).^31^ While initial photoexcitations in these materials are highly delocalized large polarons with a high mobility, within a few ps they transfer to a localized small-polaron state with significantly lower mobility.^29,30,32^ Importantly, this has been proposed as a key reason underlying the underperformance of silver–bismuth halide semiconductors.^30,33^ The low electronic dimensionality of these materials, increases in the acoustic deformation potential associated with bismuth substitution, and the softness of silver–bismuth bonds have all been proposed as factors promoting this ultrafast localization process.^33^ Therefore, to evaluate the potential of alloying strategies for silver–bismuth halides, it is imperative to investigate the effects of alloying on charge-carrier transport and its implication on the charge-carrier localization process in these materials.

This work investigates charge-carrier transport in alloyed Cs_2_AgSb_*x*_Bi_1–*x*_Br_6_ double perovskite thin films and uncovers the interplay between alloying and ultrafast charge-carrier localization. Here, inherent limitations to the alloying strategy for silver–bismuth halides emerge as a consequence of the hopping-transport regime that is typically associated with small polarons. We study a series of phase-pure Cs_2_AgSb_*x*_Bi_1–*x*_Br_6_ thin films fabricated via spin-coating. The effect of alloying on the electronic structure of Cs_2_AgSb_*x*_Bi_1–*x*_Br_6_ thin films is investigated by using reflection/transmission spectroscopy. Furthermore, we demonstrate the persistence of charge-carrier localization in Cs_2_AgSb_*x*_Bi_1–*x*_Br_6_ at different Bi/Sb concentrations by optical pump–terahertz probe (OPTP) spectroscopy. By analyzing charge-carrier mobilities, we reveal the interplay between charge-carrier localization and alloying. We observe that alloying substantially hinders the transport of localized charge carriers compared with that of delocalized charge carriers present initially after excitation. By comparing the transport trends with the changes in electronic structure and effective masses upon composition change, we ascribe this effect to different energetic landscapes probed by localized and delocalized charge carriers in alloyed semiconductors. Our findings thus suggest that the alloying approach for silver–bismuth halides has intrinsic limitations owing to the nature of charge-carrier transport in these materials.

To investigate the effect of alloying on charge-carrier transport in silver–bismuth double perovskites, we fabricated a complete series of Cs_2_AgSb_*x*_Bi_1–*x*_Br_6_ thin films with *x* = 0, 0.2, 0.4, 0.6, 0.8, and 1. With increasing antimony fraction *x* from 0 to 1, we therefore alloyed bismuth-based (Cs_2_AgBiBr_6_) double perovskites with antimony-based (Cs_2_AgSbBr_6_) double perovskites. Cs_2_AgBiBr_6_ and Cs_2_AgSbBr_6_ have a cubic *Fm*3̅*m* double perovskite (elpasolite) structure.^7,8,26^ In alloyed Sb/Bi double perovskites, [SbBr_6_]^3–^ octahedra statistically replace [BiBr_6_]^3–^ octahedra in the conventional alternating corner-sharing [AgBr_6_]^5–^ and [BiBr_6_]^3–^ network (Figure 1a). We determined the structure and phase purity of the Cs_2_AgSb_*x*_Bi_1–*x*_Br_6_ thin film series by X-ray diffraction (XRD) measurements. As shown in Figure 1b, the main XRD peaks correspond to the reference peaks for Cs_2_AgBiBr_6_ and Cs_2_AgSbBr_6_. Furthermore, the absence of split diffraction peaks in the measured diffraction patterns (Figure S1) rules out a coexistence of different phases. The extracted lattice parameters show continuously varying lattice *d*-spacing from 11.190 ± 0.006 Å for Cs_2_AgSbBr_6_ to 11.260 ± 0.002 Å for Cs_2_AgBiBr_6_ (Figure S2). This change in the cubic lattice constant is consistent with the different ionic radii of Sb^3+^ and Bi^3+^, confirming previous end point observations.^8,26^ Notably, the continuous variation of the lattice constant is in line with the formation of Cs_2_AgSb_*x*_Bi_1–*x*_Br_6_ alloys and rules out the possible segregation of Bi-rich or Sb-rich phases.

**Figure 1:**
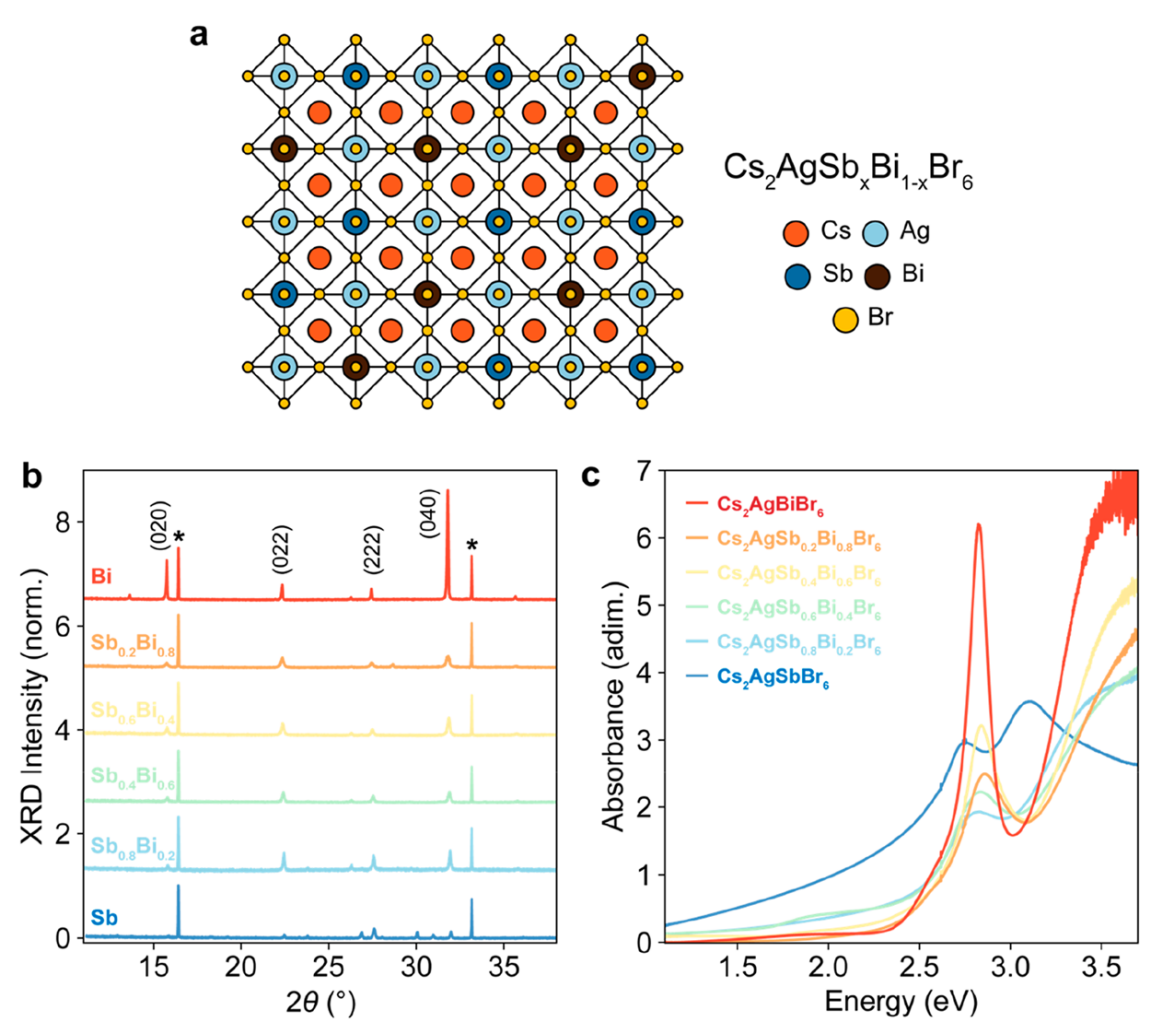
Structure and optical absorption properties of Cs_2_AgSb_x_Bi_1–*x*_Br_6_ alloyed semiconductors. (a) Schematic of Cs_2_AgSb_*x*_Bi_1–*x*_Br_6_ crystal structures. Cations and anions are represented by different colors, as shown in the legend. (b) XRD patterns of Cs_2_AgSb_*x*_Bi_1–*x*_Br_6_ thin films on quartz. Peaks assignments are reported at the top of the figure; asterisks indicate quartz peaks. (c) Absorption spectra are stitched across two ranges: one measured using a tungsten lamp–Si detector configuration for energies below 2.6 eV and another with a Xe lamp–GaP detector for energies above 2.6 eV.

In order to establish the effect of alloying on the electronic states of silver–bismuth double perovskites, we measured the UV–vis absorption spectra of the Cs_2_AgSb_*x*_Bi_1–*x*_Br_6_ thin films series in the range 1.2–3.7 eV. As shown in Figure 1c, absorption spectra for the entire thin-film series show sharp, strongly absorbing features in the ∼2.7–3.1 eV range. We observe a significant dependence of the lowest transition energy on the bismuth/antimony fraction in the Cs_2_AgSb_*x*_Bi_1–*x*_Br_6_ alloys (Figure S4), thus confirming predictions of a strong Sb/Bi alloying effect on the electronic structure of these double perovskites.^26,34^ As previously suggested by some of us and others for Cs_2_AgBiBr_6_,^10,26,30^ we attribute the sharp resonant absorption features observed for the Cs_2_AgSb_*x*_Bi_1–*x*_Br_6_ alloy series to excitonic transitions. Even though alternative attributions—for instance, to bismuth or silver intra-atomic s–p transitions^35,36^—have been proposed, recent experimental and computational results have further confirmed the excitonic nature of such absorption peaks.^33,34,37^ Specifically, first-principles methods have reconciled previous observations by demonstrating the non-hydrogenic nature of excitons in these materials.^34^ Here, deviations from the hydrogenic model arise from the electronic band structure (see Table S1 and the discussion in Supporting Note 3). The dominant orbital contribution of Sb/Bi to the conduction band minimum (CBM), and Ag to the valence band maximum (VBM), results in charge-carrier localization in distinct octahedra (i.e., electrons in [(Sb/Bi)X_6_]^3–^ and holes in [AgX_6_]^5–^). This is known as the “chemical confinement” effect, which has been demonstrated to determine anisotropic excited states with high binding energies for this family of semiconductors.^34^

Therefore, the observed bismuth–antimony fraction dependence of the measured absorption peaks originates from the different orbital contributions of bismuth and antimony to the band edges. Interestingly, the excitonic transitions for Cs_2_AgSbBr_6_ starkly differ from those we observe for the other compositions in the Cs_2_AgSb_*x*_Bi_1–*x*_Br_6_ series. While only a single excitonic peak can be seen near a photon energy ∼2.8 eV for *x* ranging from 0 to 0.8 in the Cs_2_AgSb_*x*_Bi_1–*x*_Br_6_ series, the *x* = 1 thin film (i.e., Cs_2_AgSbBr_6_) presents two excitonic peaks at ∼2.7 and ∼3.1 eV. The substantial difference between the Bi and Sb end points can be ascribed to the different energetics of Bi and Sb orbitals. Specifically, this results in the lowest direct transition for Cs_2_AgBiBr_6_ being at the X point of the Brillouin zone, ∼700 meV above the fundamental, indirect X → L gap (vide infra). Because of the strong spin–orbit splitting of Bi p orbitals at the X point (see Figure S7a), the second direct transition is far above the first one (1.37 eV). In the case of Cs_2_AgSbBr_6_, although spin–orbit splitting is negligible at the CBM (L point), it has a non-negligible effect at the X point, splitting the Sb p orbitals (see Figure S7b). In this case, the first and second direct transitions are separated only by ∼400 meV. Because the dispersion of the first and second conduction bands at the X point is similar (see discussion below), it is reasonable to assume that they would have similar exciton binding energies. Hence, we propose that the observed excitonic structure arises from direct excitonic transitions at the X point in Cs_2_AgSbBr_6_. Crucially, the similarity of the absorption spectra of alloyed Cs_2_AgSb_*x*_Bi_1–*x*_Br_6_ (*x* = 0.2–0.8) thin films to that of Cs_2_AgBiBr_6_ (i.e., a single excitonic peak at ∼2.7 eV) suggests a bismuth-like character of the CBM even at considerably high Sb fractions. This conclusion sheds further light on the type II staggered band alignment for the Cs_2_AgSb_*x*_Bi_1–*x*_Br_6_ series, proposed by Hoye, Walsh, and co-workers.^26^ While indirect type II absorption transitions cannot contribute significantly to the direct absorption transitions discussed here, we note that our observation of a bismuth-like CBM character supports the hypothesis of nonlinear mixing of electronic states proposed by Hoye, Walsh, and co-workers for the indirect gap.^26^

Having assessed changes induced by antimony/bismuth alloying to the electronic band structure, we investigated their implications on charge-carrier transport in Cs_2_AgSb_*x*_Bi_1–*x*_Br_6_ thin films. We studied the charge-carrier dynamics and mobility by using OPTP spectroscopy. In OPTP experiments, we photoexcited charge carriers by using 3.1 eV pulses and monitored the fractional transmission (Δ*T*/*T*) of THz pulses, proportional to the photoconductivity defined as Δσ = *ne*μ̃. As discussed in Supporting Note 2, the observed photoconductivity signal is proportional to the photon-to-charge branching ratio ϕ (i.e., the fraction of free electron and hole density generated per absorbed photon density), and therefore charge-carrier mobilities extracted here are effective electron–hole sum mobilities ϕμ.

Figure 2a shows OPTP transients measured for a subset of Cs_2_AgBiBr_6_, Cs_2_AgSb_0.4_Bi_0.6_Br_6_, and Cs_2_AgSbBr_6_ films, whereas measurements for the entire series are shown in Figure S5. As previously demonstrated by Wright et al. for Cs_2_AgBiBr_6_,^30^ we note that the observed OPTP transients are representative of free charge-carrier conductivities in the materials. To further confirm this, we measured photoconductivity spectra for the entire thin-film series, recorded at the photoconductivity maximum (see the inset of Figure 2a and Figure S6). Even though we expect the coexistence of exciton and free carrier populations following the initial photoexcitation (vide infra), photoconductivity spectra in the measured range (0.5–2.5 THz) are consistent with a free-charge-carrier conductivity signal.^38^ Here, the absence of exciton signatures in the measured range is caused by the high exciton binding energy in the materials (i.e., shifting excitonic resonances outside of the measured THz range) and implies that the measured OPTP signal can be used to selectively monitor the free carrier population. As shown in the inset of Figure 2a, we observe small deviations from the ideal Drude behavior (i.e., a flat real part and a zero imaginary part of the photoconductivity), which are well described by the phenomenological Drude–Smith model (see the Supporting Information). As previously demonstrated for other materials, these deviations arise from backscattering and charge-carrier localization effects and are consistent with the “chemical confinement” effect expected for these materials, already reported for other silver–bismuth materials.^29,31,34^

**Figure 2:**
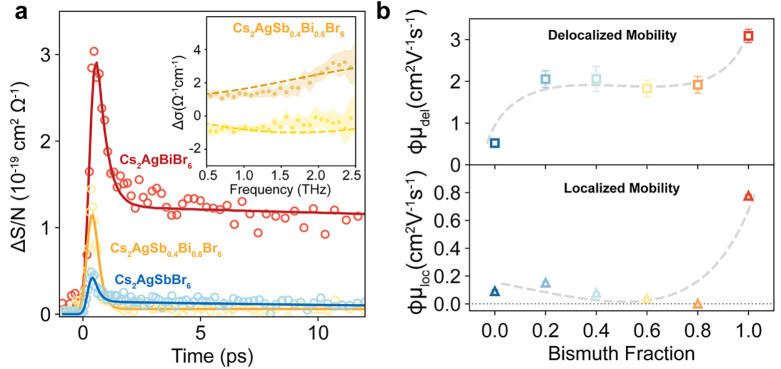
Impact of alloying in Cs_2_AgSb_x_Bi_1–*x*_Br_6_ on charge-carrier transport. (a) Photoinduced THz sheet conductivity for Cs_2_AgBiBr_6_ (red), Cs_2_AgSb_0.4_Bi_0.6_Br_6_ (yellow), and Cs_2_AgSbBr_6_ (blue) thin films, measured after 3.1 eV pulsed excitation at a fluence of 80 μJ cm^–2^, normalized by the areal charge-carrier density *N*. Open circles are experimental data, and solid lines represent fits to the two-level mobility model described in Supporting Note 2. Inset: real (dark yellow) and imaginary (light yellow) parts of the photoinduced THz conductivity spectra for Cs_2_AgSb_0.4_Bi_0.6_Br_6_ thin films, measured at the photoconductivity maximum (*t* = 1 ps) following 3.1 eV excitation at a fluence of 80 μJ cm^–2^. Full circles represent experimental data (with shaded areas indicating statistical error), whereas dashed lines are fits according to the Drude–Smith model (Supporting Information). (b) Effective THz electron–hole sum mobilities for Cs_2_AgSb_*x*_Bi_1–*x*_Br_6_ thin films plotted as a function of the bismuth fraction (1 – *x*), extracted from the two-level mobility model. Dashed lines are guides for the eye.

By analyzing OPTP transients for the Cs_2_AgSb_*x*_Bi_1–*x*_Br_6_ thin films series (Figure S5), we consistently observe an ultrafast photoconductivity decay in the first few picoseconds following photoexcitation and a subsequent plateauing of the photoconductivity. Some of us recently reported similar ultrafast decay of the photoconductivity for various bismuth- and silver–bismuth-based semiconductors (e.g., Cs_2_AgBiBr_6_, Cu_2_AgBiI_6_, (4F-PEA)_4_AgBiI_8_, NaBiS_2_, AgBiS_2_, Cu_4*x*_(AgBi)_1–*x*_I_4_).^30−32,39−43^ A variety of excited state processes (e.g., intrinsic self-trapping, charge-carrier trapping at extrinsic defects, exciton formation, and charge-carrier cooling) could, in principle, yield such ultrafast photoconductivity decays. However, as discussed extensively by Wright et al. and Buizza et al. for Cs_2_AgBiBr_6_ and Cu_2_AgBiI_6_, the fluence-independent behavior of the observed decay and the associated change in transport regime (from bandlike to thermally activated transport; see below) observed in temperature-dependent OPTP experiments strongly suggest that such decay derives from ultrafast formation of small polarons in Cs_2_AgBiBr_6_—namely, an ultrafast localization of the charge carriers caused by a significant distortion of the lattice.^30,32^

To quantify the ultrafast localization process, we fitted the fluence-dependent OPTP data to the two-level mobility model developed by Wright et al. and Buizza et al. (Figure S5 and Supporting Note 2).^29,30,32^ This model assumes two photoexcited states: an initially photogenerated delocalized state with high mobility (μ_del_) and a subsequently formed, localized state with low mobility (μ_loc_). Therefore, the observed sheet photoconductivity can be interpreted as the sum of contributions from these states Δ*S* = *e*(*N*_loc_μ_loc_ + *N*_del_μ_del_), where *N*_del_ and *N*_loc_ are the charge-carrier density per units of area for the delocalized and localized state, respectively. Here, we note the excellent agreement between experimental data and two-level mobility model fits and the fluence-independent nature of observed dynamics (Figure S5), which further confirms the presence of an ultrafast localization process in these materials. However, as shown in Figure 2a, the comparison between different Cs_2_AgSb_*x*_Bi_1–*x*_Br_6_ thin films reveals a significant dependence on the composition for both initial and long-time sheet photoconductivity. These differences were confirmed by ϕμ_del_ and ϕμ_loc_ parameters extracted from the two-level mobility model (Figure 2b). We observe the highest effective mobilities in the series for Cs_2_AgBiBr_6_, reaching ϕμ_del_ ≈ 3 cm^2^ V^–1^ s^–1^ and ϕμ_loc_ ≈ 0.8 cm^2^ V^–1^ s^–1^, a value similar to that reported previously by Wright et al.^30^ Increasing antimony fractions (thus, decreasing bismuth fractions) result in lower mobilities, with alloyed thin films (*x* = 0.2–0.8) showing a slightly reduced delocalized effective mobility ϕμ_del_ ≈ 2 cm^2^ V^–1^ s^–1^ and a significantly reduced localized effective mobility in the range of 0.1–0.2 cm^2^ V^–1^ s^–1^ and Cs_2_AgSbBr_6_ the lowest delocalized effective mobility of ϕμ_del_ ≈ 0.5 cm^2^ V^–1^ s^–1^ within the series and similarly low localized effective mobility of ≈0.1 cm^2^ V^–1^ s^–1^.

Observed trends in the delocalized mobilities, associated with the initially formed large polarons, are likely to be determined by several complementary effects, including both intrinsic (i.e., electronic band structure, charge-carrier couplings, and excitonic effects) and extrinsic (i.e., crystallinity, grain boundary scattering, and generally disordered electronic landscape). To quantify how electronic band structure changes between Cs_2_AgBiBr_6_ and Cs_2_AgSbBr_6_ can influence charge-carrier mobilities, we performed first-principles calculations of the effective-mass tensors and anisotropies. Our periodic density functional theory (DFT) calculations in VASP^52^ employed the Perdew–Burke–Ernzerhof (PBE) exchange-correlation functional^53^ and accounted for SOC to describe the band structure features of Cs_2_AgBiBr_6_ and Cs_2_AgSbBr_6_ (Figure 3). As explained in detail in Supporting Note 3, we relaxed the atomic positions and lattice constants, which resulted in good agreement with the experimental findings (Table S2). The relaxed structures were used to compute the band structures and effective mass tensors, which were evaluated in uniform grids around the band edges. The anisotropies were calculated from the eigenvectors of the effective mass tensors. As reported in Table 1, the conductivity effective masses *m*_e_* (calculated as the harmonic mean of effective masses across different directions; see Supporting Note 3) of Cs_2_AgSbBr_6_ are lower than those of Cs_2_AgBiBr_6_. This difference is stronger for the electron effective masses, as expected from the dominant Bi/Sb orbital contribution to the CBM. Interestingly, replacing Bi with Sb lowers the degree of anisotropy β of electrons but increases the anisotropy for holes. According to the Drude model, the charge-carrier mobility is inversely proportional to the effective mass m*, i.e., μ = *e*τ/m*, where τ is the scattering time. Because introducing Sb reduces the effective masses, the observed lower mobilities for Sb-containing compounds cannot be explained directly by changes in the electronic band structure, as they would predict higher mobilities. Rather, they must originate from other intrinsic or extrinsic effects.

**Table 1:** Electron and Hole Conductivity Effective Masses m* (in Units of Electron Rest Mass *m*_o_) and Degree of Anisotropy β of Cs_2_AgBiBr_6_ and Cs_2_AgSbBr_6_

	*m*_e_^*^	β_e_	*m*_h_^*^	β_h_
Cs_2_AgBiBr_6_	0.33	0.13	0.35	0.58
Cs_2_AgSbBr_6_	0.27	0.05	0.32	0.69

**Figure 3:**
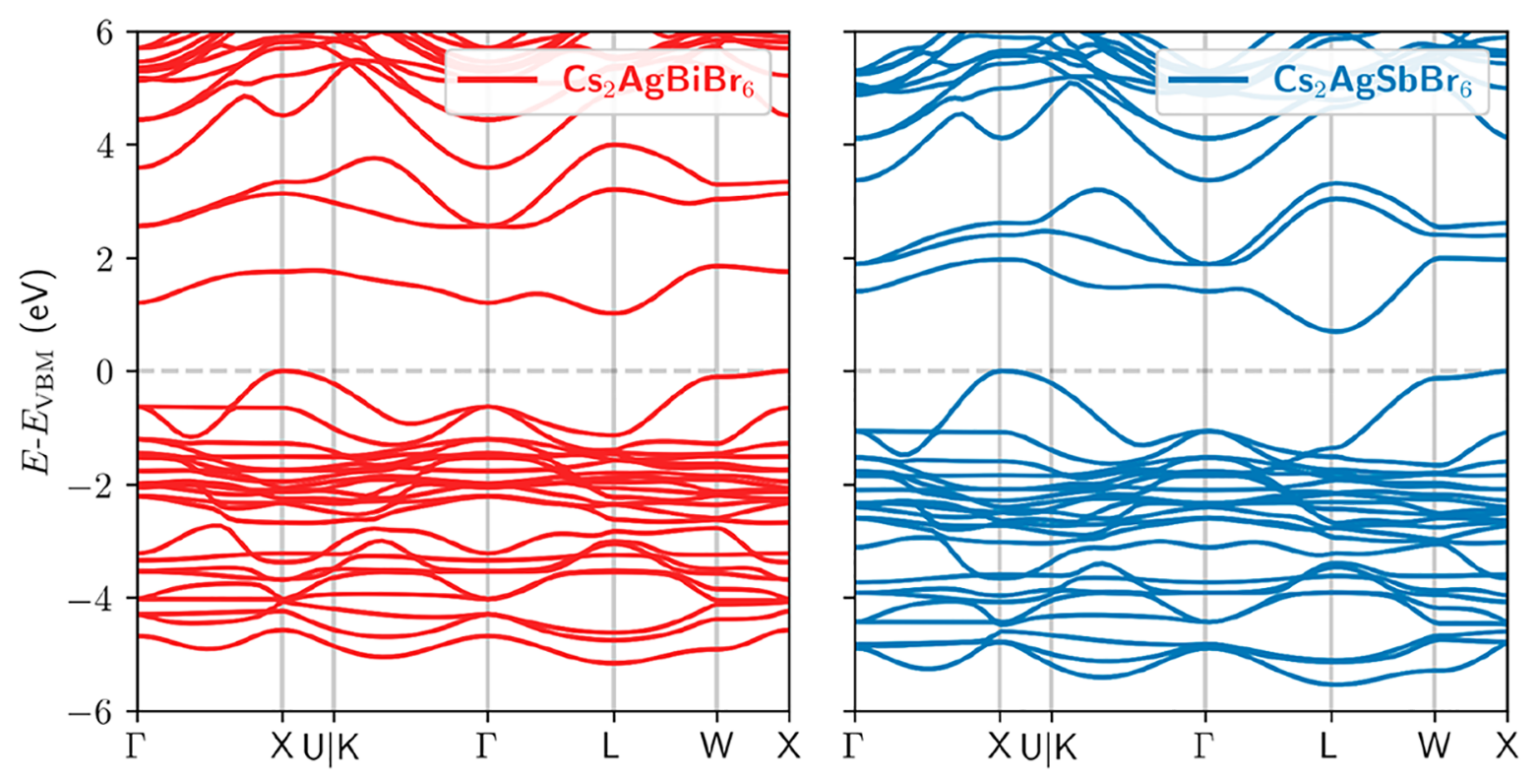
DFT band structure calculation for Cs_2_AgBiBr_6_ and Cs_2_AgSbBr_6_. Electronic band structures of Cs_2_AgBiBr_6_ (left panel) and Cs_2_AgSbBr_6_ (right panel) as calculated by using DFT. Zero energy corresponds to the top of the valence band. See Supporting Note 3 for details of calculations.

To explore alternative origins of these mobility trends, we note that as discussed in Supporting Note 1, grain sizes between Cs_2_AgBiBr_6_ (≈110 nm) and Cs_2_AgSbBr_6_ (≈80 nm) thin films appear to differ only slightly. Considering that THz photoconductivity typically probes intragrain mobilities on the 10 nm length scale in these materials, we believe these differences cannot fully account for the observed 6-fold reduction in mobility for the Cs_2_AgSbBr_6_ phase. Therefore, we posit that dielectric and excitonic effects could play a role in the observed mobility trends. As discussed for the absorption spectra, it is widely accepted that double halide perovskites are strongly excitonic systems.^30,39,44,45^ Crucially, Biega et al. calculated a higher exciton binding energy for Cs_2_AgSbBr_6_ (≈250 meV) than for its bismuth counterpart Cs_2_AgBiBr_6_ (≈180 meV).^34^ By applying the Saha equation (Supporting Note 4), we estimated a significantly reduced fraction of free charge carriers from ϕ ≈ 0.4 for Cs_2_AgBiBr_6_ to ≈0.1 for Cs_2_AgSbBr_6_. Although we cannot directly quantify the photon-to-charge-carrier branching ratio ϕ based on the fraction of free charge carriers α estimated from the Saha equation, these simulations confirm that excitonic effects are more significant for the antimony compound and could thus play a role in determining reduced branching ratio ϕ values and therefore yield lower effective mobility values ϕμ.

We further discuss the impact of alloying on localized-state mobilities in Cs_2_AgSb_*x*_Bi_1–*x*_Br_6_ thin films, attained after the initial picosecond relaxation process. As shown in Figure 2c, extracted localized-state mobilities show a divergent trend with respect to delocalized-state mobilities. Namely, lower ϕμ_loc_ values are observed for intermediate concentrations, and *x* = 0.6 and 0.8 show negligible localized-state mobility, below the detection limit of our instrument (>0.05 cm^2^ V^–1^ s^–1^). Considering the significant differences in initial mobilities, we isolated the effect of alloying on the ultrafast localization process by calculating the fraction of retained mobility after the localization process, defined as *p* = μ_loc_/μ_del_. This “mobility retention” ratio (Figure 4a) shows that localization effects are less severe in the unalloyed Cs_2_AgBiBr_6_ (*p* ∼ 25%) and Cs_2_AgSbBr_6_ (*p* ∼ 18%) thin films, while *p* values well below 10% are observed for alloyed samples. Furthermore, the fraction of retained mobility approaches zero for Cs_2_AgSb_0.2_Bi_0.8_Br, for which the localized mobility is below our detection limit. Notably, the divergence in mobility trends between localized and delocalized states with alloy composition and the opposing trends of exciton binding energy reported by Biega et al.^34^ (see Supporting Note 3) indicate that the effects of alloying on charge-carrier transport cannot be well explained by modulations in the electronic band structure and excitonic/dielectric effects.

**Figure 4:**
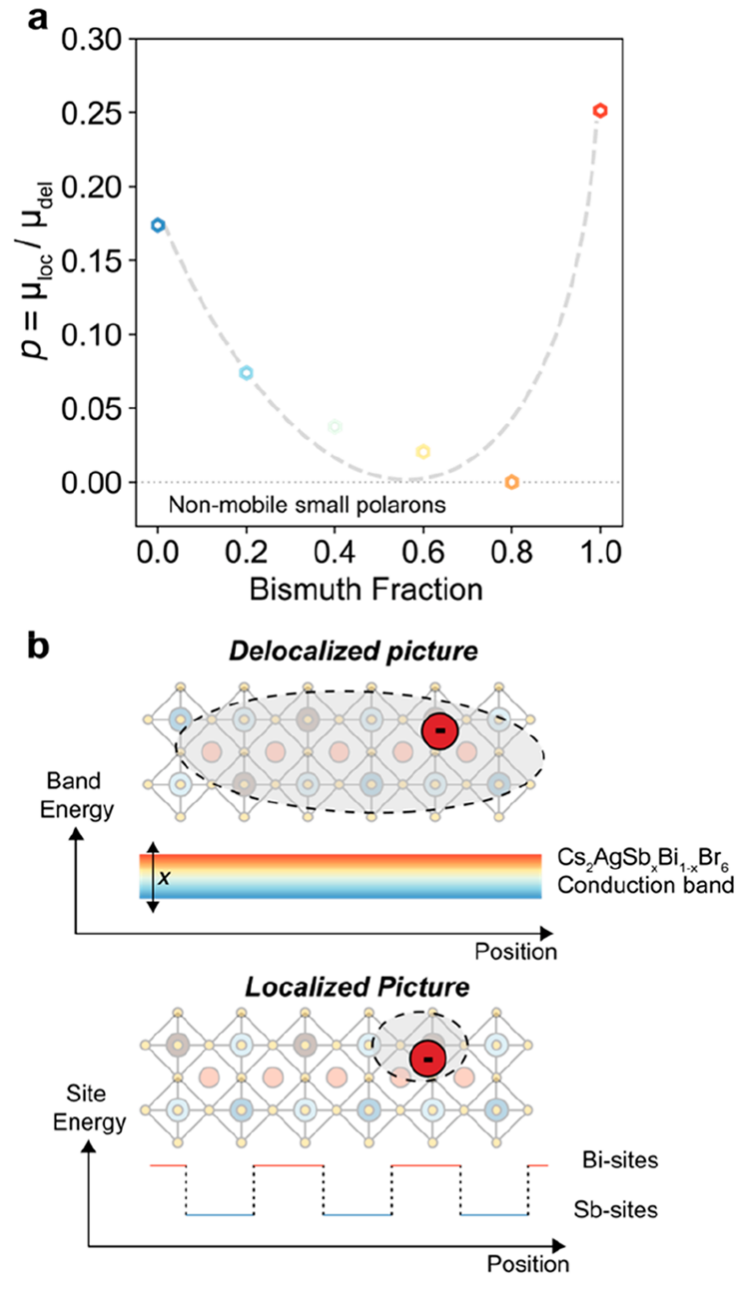
Effect of alloying on localized-state mobility. (a) Mobility retention ratio *p* calculated as the ratio between localized and delocalized mobility values, plotted as a function of the bismuth fraction (1 – *x*) for the Cs_2_AgSb_*x*_Bi_1–*x*_Br_6_ series. The dashed line is a guide for the eye. (b) Schematic illustration of the different electronic landscapes probed by delocalized and localized charge carriers in alloyed Cs_2_AgSb_*x*_Bi_1–*x*_Br_6_ thin films. Delocalized charge carriers probe the electronic bands (in this illustration, the conduction band), whose energetics can be tuned with the composition of the alloys. Localized charge carriers may hop between Bi and Sb sites, thus increasing the probability of being trapped at Sb sites.

Here, the different transport regimes associated with μ_del_ and μ_loc_ are key to understanding the effects of alloying on localized-state transport. As already reported for several silver–bismuth semiconductors,^29−31^ the initial ultrafast localization process is associated with a significant change in the transport regime. While initially generated delocalized large polarons generally exhibit band-like transport behavior, the subsequently formed small polarons display typical temperature-activated hopping transport.^29−31,46,47^ We posit that this change in the transport regime also determines how charge carriers probe the disordered energetic landscape in these materials. As summarized in Figure 4b, the delocalized wave functions of large polarons are able to probe the electronic bands formed upon alloying. Thus, within the limits defined by percolation theory developed by Efros and Shklovskii,^48^ the effect of alloying on charge-carrier mobilities for delocalized states (discussed above) is mainly determined by the different orbital contributions to the electronic band structure. Even though enhanced potential scattering was reported to compound charge-carrier scattering in alloys,^49^ such alloy-scattering contributions were demonstrated to be marginal.^50,51^ On the other hand, localized small polarons, because of their contracted wave function, will probe the local lattice sites associated with either bismuth or antimony orbitals. In this case, the type II heterojunction alignment between Cs_2_AgBiBr_6_ and Cs_2_AgSbBr_6_ is likely to cause Sb sites to act as traps,^26^ thus transforming itinerant into nonmobile small polarons and reducing their mobility.

In conclusion, our results unravel the inherent limitations faced by alloying strategies for silver–bismuth double halide perovskites with potential implications for the entire family of silver–bismuth semiconductors. While bismuth/antimony alloying proves successful in tuning the electronic band structure, thereby allowing us to devise bandgap engineering strategies, it also dramatically affects charge-carrier transport in these semiconductors. Our investigation of the photoconductivity in Cs_2_AgSb_*x*_Bi_1–*x*_Br_6_ thin films provides unequivocal evidence of reduced charge-carrier mobilities as a result of alloying. We demonstrate that the ultrafast charge-carrier localization process, previously demonstrated for Cs_2_AgBiBr_6_, underlies this unexpected reduction in the mobility of localized states. On the one hand, initially delocalized charge carriers show mobility trends compatible with the observed changes in electronic band structure and dielectric environment. On the other hand, mobilities for rapidly formed localized charge carriers are significantly reduced upon alloying. We conclude that the formation of small-polaron states reshapes the “effective” energy landscape probed by charge carriers and promotes their localization at lower-energy Sb sites. Crucially, our findings further raise the urgency of developing new approaches to prevent, or at least mitigate, the ubiquitous localization of charge carriers in silver–bismuth-based semiconductors, thus unleashing the full potential of alloying strategies. On a more fundamental level, our work provides insights into charge-carrier dynamics of polaronic materials and highlights how a nominally identical electronic landscape may be experienced very differently by delocalized and localized charge carriers. Our findings shed new light on the inherent limitations of alloying strategies for the silver–bismuth semiconductor family and provide insights to guide innovative strategies for designing new semiconductors for renewable energy applications.

## Supporting Information Available

Additional details on experimental procedures, materials, and methods; analysis of X-ray diffraction patterns, fluence-dependent OPTP, and photoconductivity spectra for the Cs_2_AgSb_*x*_Bi_1–*x*_Br_6_ thin film series; additional details on the derivation of charge-carrier mobility from OPTP measurements; and additional details on first-principles calculations (PDF)
