# Supplementary material for: Alloying Effects on Charge-Carrier Transport in Silver–Bismuth Double Perovskites
Source: J Phys Chem Lett. 2023 Nov 10;14(46):10340–7. doi: 10.1021/acs.jpclett.3c02750 (PMC10683067; doi:10.1021/acs.jpclett.3c02750)
Supplement: Supplementary file 1 — jz3c02750_si_001.pdf [file jz3c02750_si_001.pdf]

Supplementary Information for

Alloying Effects on Charge-Carrier Transport in Silver-Bismuth Double Perovskites

*Marcello Righetto,[1] Sebastián Caicedo-Dávila,[2] Maximilian T. Sirtl,[3] Vincent J.-Y. Lim,[1] Jay B. Patel,[1] David A. Egger,[2] Thomas Bein,[3] Laura M. Herz[1,4]**

[1.] Department of Physics, University of Oxford, Clarendon Laboratory, Parks Road, Oxford, OX1 3PU, United Kingdom

[2.] Physics Department, TUM School of Natural Sciences, Technical University of Munich, James-Franck-Straße 1, Garching, 85748 Germany

[3.] Department of Chemistry and Center for NanoScience (CeNS), University of Munich (LMU), Butenandtstr. 11, 81377 Munich, Germany

[4.] Institute for Advanced Study, Technical University of Munich, Lichtenbergstrasse 2a, D-85748 Garching, Germany

*Corresponding Author's Email: laura.herz@physics.ox.ac.uk

Materials & Methods

Thin Film Preparation

The stock solution was prepared by dissolving CsBr (Alpha Aesar, 99.999 % metals basis), $BiBr_3$ (Alpha Aesar, 99.9 % metals basis) and AgBr (Alpha Aesar, 99.998 % metals basis) and $SbBr_3$ (Alpha Aesar, 99.5 % metals basis) in 1 mL DMSO (Sigma Aldrich, anhydrous, ≥99.9 %) by vigorous stirring at 130 °C for 60 minutes to obtain a 0.5 M solution. All Steps were performed in a nitrogen-filled glovebox with controlled atmosphere.

The substrates were cleaned with a detergent (Hellmanex), followed by washing with acetone and ethanol and dried under an air stream. Afterwards, the substrates were cleaned with oxygen plasma for 5 minutes and immediately transferred into the glovebox. Prior to the spincoating step, the substrates and the solution were placed on a hotplate (Heidolph with internal temperature sensor) at temperatures between 50 °C ($Cs_2AgBi_{1-x}Sb_xBr_6$ solutions) and 60 °C (pure $Cs_2AgBiBr_6$ solution) to be preheated. The stock solution was constantly stirred. The thin films were fabricated by spincoating the warm solution dynamically (1000 rpm for 10 s, followed by a second step at 6000 rpm for 35 seconds) onto the preheated substrates (70 µL of the solution were dropped immediately after the substrate started to spin at 1000 rpm).

After the spincoating, the thin films were annealed at different temperatures and times, dependent on the stoichiometry. For $Cs_2AgSbBr_6$, $Cs_2AgSb_{0.8}Bi_{0.2}Br_6$, $Cs_2AgSb_{0.6}Bi_{0.4}Br_6$, $Cs_2AgSb_{0.4}Bi_{0.6}Br_6$, and $Cs_2AgSb_{0.2}Bi_{0.8}Br_6$, preheating was set at 50 °C and thin films were annealed at 135 °C for 20 minutes. For $Cs_2AgBiBr_6$ preheating was set at 60 °C and thin films were annealed at 275 °C for 5 minutes.

Absorption Measurements

UV-Visible-NIR absorption spectra were measured using a Bruker-Vertex 80v Fourier-Transform Infrared (FTIR) spectrometer fitted with a transmission/reflection accessory. Spectra in the region 1.2 – 2.6 eV were taken by using a tungsten halogen source and Si detector, and spectra in the region 2.6 – 3.7 eV were taken by using a tungsten halogen source and a Si detector. The two datasets obtained were stitched by matching the absorbance curves at 2.6 eV.

X-ray Diffraction

X-ray Diffraction (XRD) patterns were measured using a PANalytical X'Pert powder diffractometer, using radiation from a Cu K-α_1 source, across 2θ values ranging from 5 – 45°. The scan speed was 0.01 °/s and the step size was set to 0.004 °. In order to correct against sample tilt, the z-cut quartz peak at 2θ = 16.43° was used as a reference to pin the patterns measured on quartz substrates.

Optical-Pump-Terahertz-Probe Spectroscopy

We performed optical pump terahertz probe (OPTP) experiments by using a setup described in detail elsewhere.[1] In brief, an amplified Ti:sapphire laser system (Spectra-Physics Spitfire) provides 800-nm light pulses with 5-kHz repetition rate and 35-fs pulse duration. Using this fundamental output, single-cycle THz radiation pulses are generated in a spintronic emitter (W/$Co_{40}Fe_{40}B_{20}$/Pt multilayer film on quartz) via the inverse spin Hall effect.[2] Furthermore, samples are excited by 400-nm pulses, generated by second-harmonic generation in a beta-barium-borate (BBO) crystal. Fractional changes in the THz transmission in the range 0.5-2.5 THz following photoexcitation are measured by using free-space electro-optic (EO) sampling. Detection of the THz pulses is performed using electro-optic sampling in a 1-mm-thick (110)-

ZnTe crystal. We measured $Cs_2AgSb_xBi_{1-x}Br_6$ thin films deposited onto 2 mm thick z-cut quartz. During OPTP measurements, the THz emission and detection optics and samples are kept under vacuum at pressures below 0.1 mbar.

Supporting Note 1: Analysis of X-ray Diffraction Patterns

As described in the Main Text, we determined the phase purity and structure of the $Cs_2AgSb_xBi_{1-x}Br_6$ thin films through XRD measurements. XRD patterns reported in the Main Text (Figure 1b) confirm the double perovskite structure for all the thin films in the studied series. In agreement with previous reports (References [3-4]), we assigned all the main XRD peaks according to the reference patterns found in Refs. [4-5].

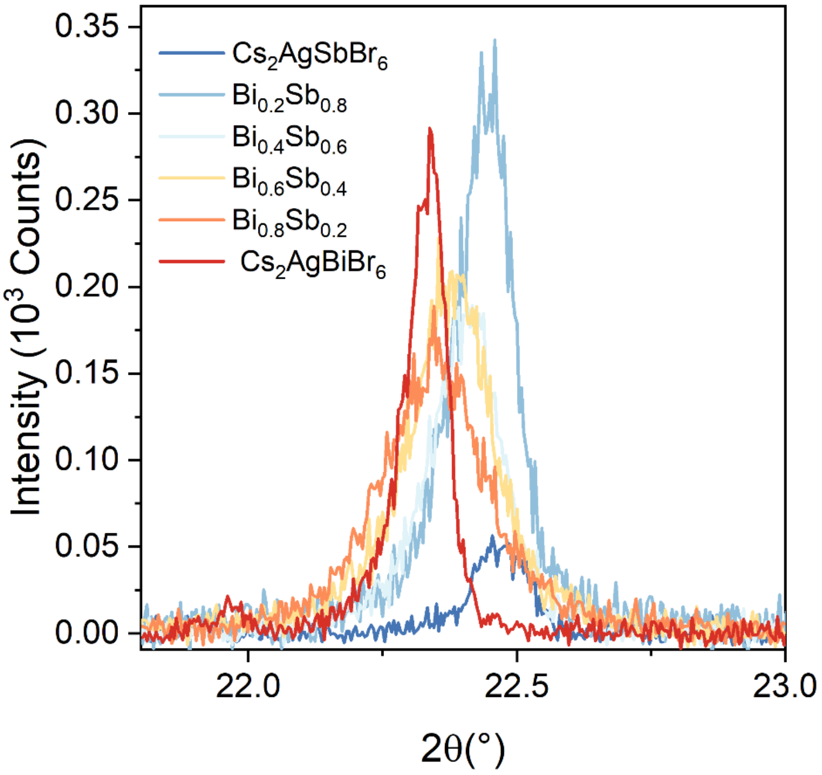

Figure S1 | X-ray diffraction peak shifts in the $Cs_2AgSb_xBi_{1-x}Br_6$ thin film series. Shift in the position of (022) diffraction peaks measured for the $Cs_2AgSb_xBi_{1-x}Br_6$ thin film series.

As shown in Figure S1, we observed a continuous shift in the main XRD peaks from larger to smaller 2θ values with increasing bismuth content. A similar peak shift could be observed for all the main XRD peaks in the measured diffractograms. These shifts are consistent with the

different ionic radii of Sb^{3+} (smaller) and Bi^{3+} (larger), which underlie the differences in lattice constant previously reported for $Cs_2AgSbBr_6$ [4] and $Cs_2AgBiBr_6$ [5]. To confirm the presence of alloying, we estimated lattice constants from the position of four XRD peaks (020), (022), (222), (040). The lattice constant values are found to increase continuously with increasing bismuth content between the endpoints: 11.190±0.006 Å for $Cs_2AgSbBr_6$ and 11.260±0.002 Å for $Cs_2AgBiBr_6$, and consistently with previous reports by Hoye and coworkers.[3]

Altogether, these continuous shifts in the lattice constant (Figure S2) and the absence of noticeable peak splittings (Figure S1) confirm homogeneous alloying between $Cs_2AgSbBr_6$ and $Cs_2AgBiBr_6$ and the absence of phase segregation.

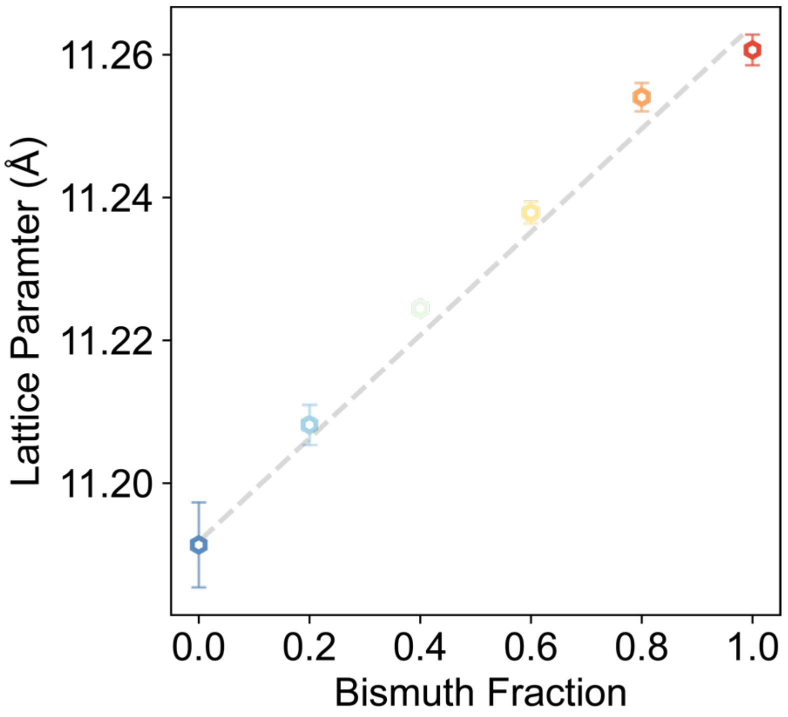

Figure S2 | Calculated lattice constant for the $Cs_2AgSb_xBi_{1-x}Br_6$ thin film series. Cubic lattice constant estimated using Bragg's law for the $Cs_2AgSb_xBi_{1-x}Br_6$ thin film series plotted as a function of the bismuth content. Reported values are the average between lattice constants estimated for four different XRD reflections (020), (022), (222), (040). Error bars represent the associated standard deviation. Dashed line is a guide to the eye.

Despite the absence of noticeable peak splitting, we observed differences in the full width at half maximum (FWHM) for the peaks. Similar effects of the XRD peak widths have been

reported by Hoye and coworkers and have been attributed to differences in the grain sizes. To confirm this, we estimated grain sizes for different compositions by using the Scherrer equations. As shown in Figure S3, we observe larger FWHM for alloyed samples, corresponding to smaller grain sizes.

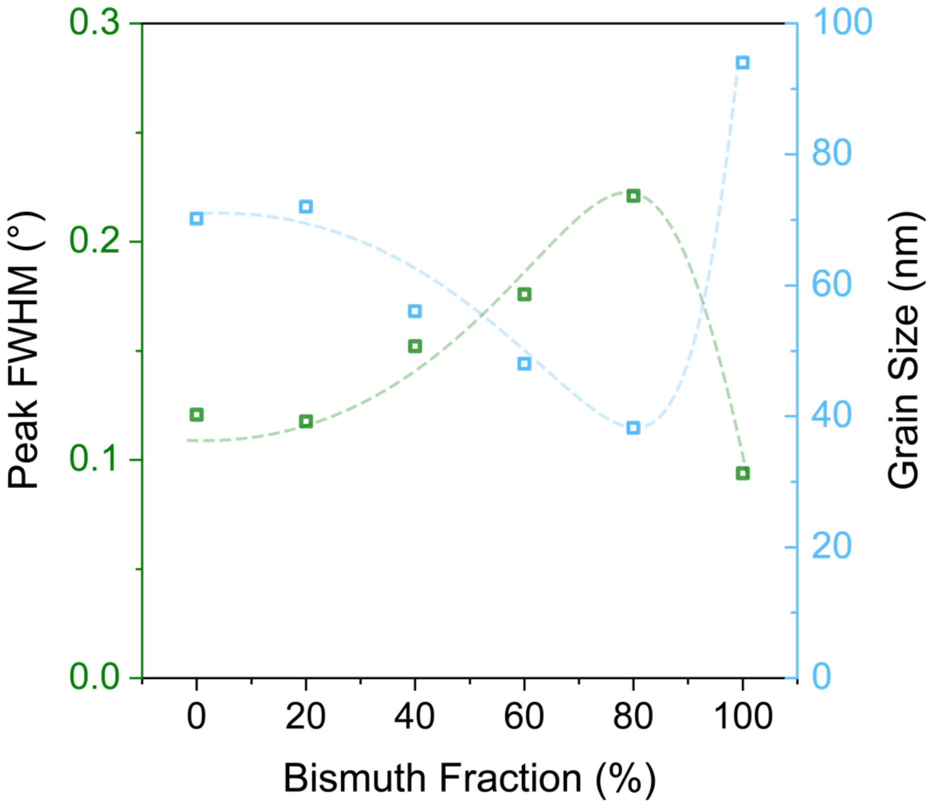

Figure S3 | Grain sizes for the $Cs_2AgSb_xBi_{1-x}Br_6$ thin film series. FWHM of the (022) diffraction peak (green squares) and estimated grain sizes (cyan squares) calculated using the Scherrer equation for different compositions of the $Cs_2AgSb_xBi_{1-x}Br_6$ thin film series. Dashed lines are a guide to the eye.

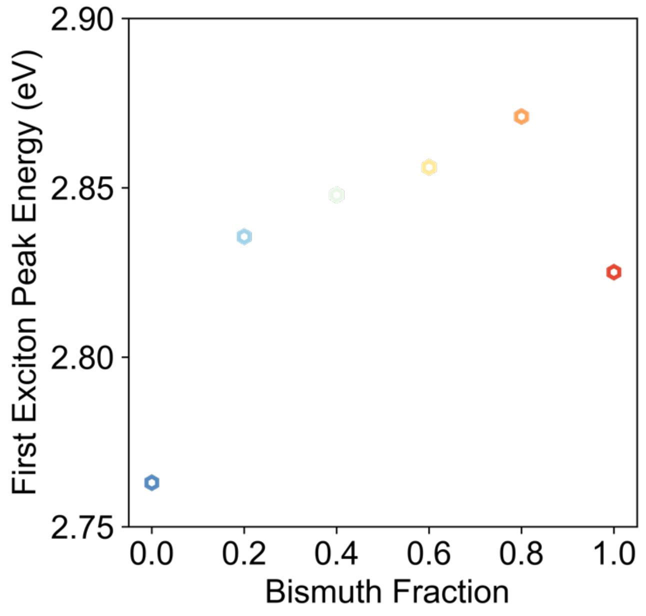

Figure S4 | Energy of the first exciton peak for the $Cs_2AgSb_xBi_{1-x}Br_6$ thin film series. Energies of the lowest exciton transition, obtained by Gaussian fitting of the lowest peak in the absorption spectra shown in Figure 1 of the Main Text, and plotted as a function of the bismuth fraction on the trivalent metal site.

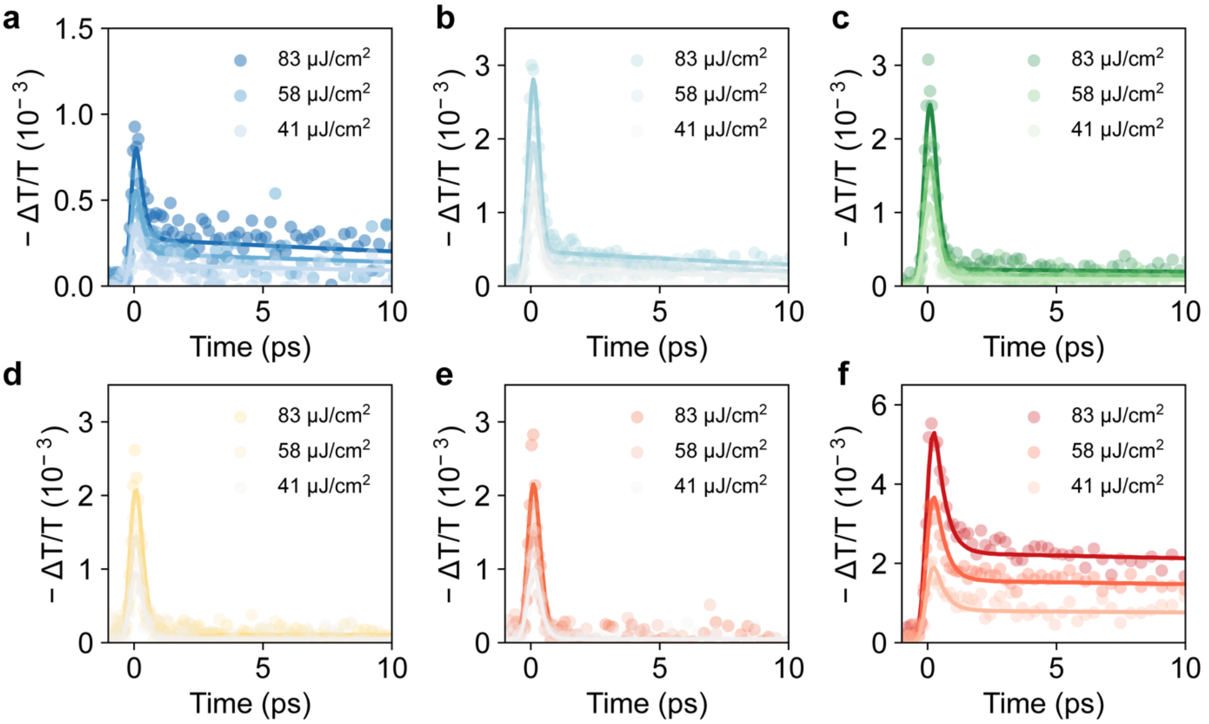

Figure S5 | Fluence-dependent optical-pump-THz-probe (OPTP) measurements. Fluence-dependent photoconductivity transients measured for (**a**) $Cs_2AgSbBr_6$, (**b**) $Cs_2AgSb_{0.8}Bi_{0.2}Br_6$, (**c**) $Cs_2AgSb_{0.6}Bi_{0.4}Br_6$, (**d**) $Cs_2AgSb_{0.4}Bi_{0.6}Br_6$, (**e**) $Cs_2AgSb_{0.2}Bi_{0.8}Br_6$, and (**f**) $Cs_2AgBiBr_6$ thin films after 3.1-eV pulsed excitation. Solid lines represent fits to the two-level mobility model. Open circles are experimental data, and solid lines represent fits to the two-level mobility model.

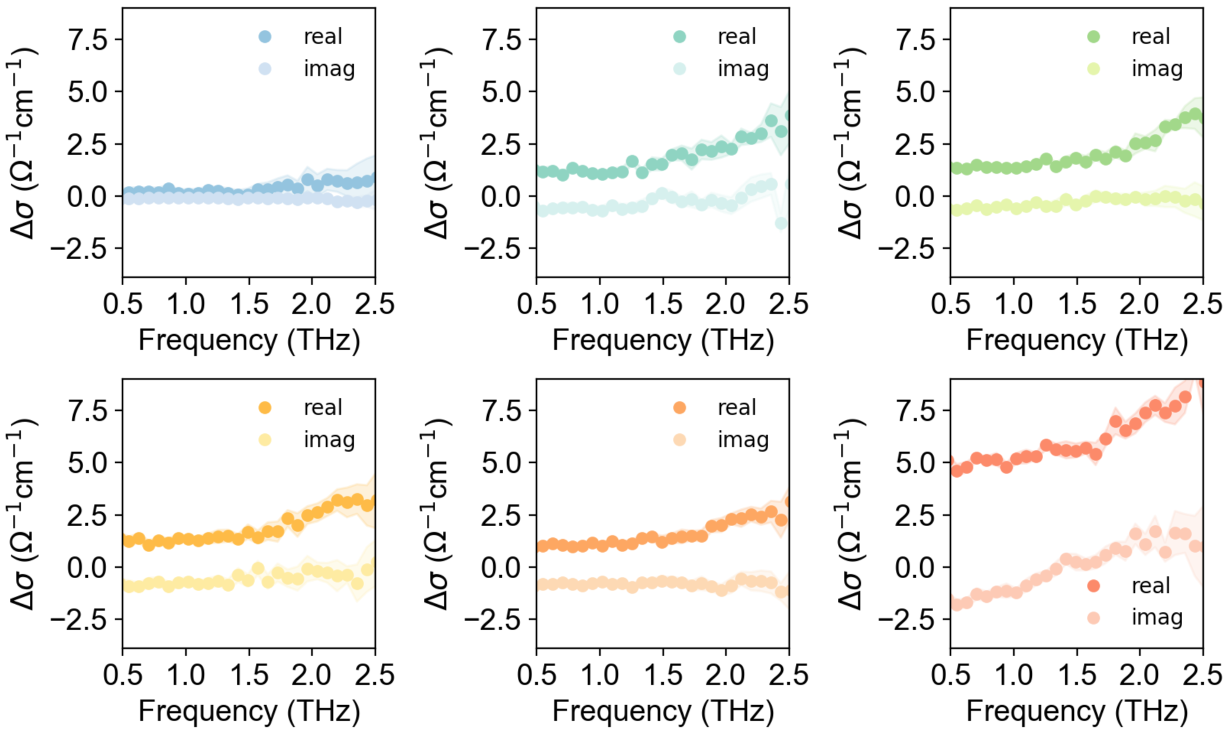

Figure S6 | Photoconductivity spectra for the $Cs_2AgSb_xBi_{1-x}Br_6$ thin film series. Terahertz photoconductivity spectra measured in the range 0.5-2.5 THz for (**a**) $Cs_2AgSbBr_6$, (**b**) $Cs_2AgSb_{0.8}Bi_{0.2}Br_6$, (**c**) $Cs_2AgSb_{0.6}Bi_{0.4}Br_6$, (**d**) $Cs_2AgSb_{0.4}Bi_{0.6}Br_6$, (**e**) $Cs_2AgSb_{0.2}Bi_{0.8}Br_6$, and (**f**) $Cs_2AgBiBr_6$ thin films. Spectra were measured in correspondence of the photoconductivity peak at a fluence of 83 μJ cm^{-2}. Dark and light dots represent the experimental data for the real and imaginary part of the photoconductivity, respectively. The shaded area indicates the standard deviation of the measured signal and give an estimate of the associated error.

Supporting Note 2: Derivation of charge-carrier mobility from OPTP measurements

We extracted effective charge-carrier mobility by fitting OPTP transients with a two-level mobility model developed by Wright et al. and Buizza et al..[1, 6]. The adopted method is based on the approach developed by Wehrenfennig et al.[7] Here, for materials with thicknesses smaller than the wavelength of the incident THz radiation, the sheet photoconductivity can be derived from the fractional change in the transmitted THz electric field $\Delta T/T$, and can be expressed as

$$\Delta S = -\epsilon_0 c(n_1 + n_3)\left(\frac{\Delta T}{T}\right) \quad \text{(S1)}$$

where $n_3 = 2.13$ and $n_1 = 1$ are the refractive indexes of quartz and vacuum, respectively.[8] Here, in order to derive the effective charge-carrier mobility from the sheet photoconductivity, the number of initially photogenerated carriers is defined as

$$N = \phi \frac{E\lambda}{hc}\left(1 - R_{pump} - T_{pump}\right) \quad \text{(S2)}$$

Where ϕ is the photon-to-charge branching ratio (i.e., the fraction of generated charges per absorbed photon absorbed), E is the pump pulse energy, λ is the excitation wavelength, and R_{pump} and T_{pump} are the reflectance and transmittance of the sample at the excitation wavelength (400 nm, 3.1 eV). Here, Equation S1 and Equation S2 can be used to extract the charge-carrier mobility μ through the relation

$$\mu = \frac{\Delta S\, A_{eff}}{Ne} \quad \text{(S3)}$$

Where A_{eff} is the effective overlap area between THz and pump beam and e is the elementary charge. Substituting Equation S1 and Equation S2 into Equation S3, we obtain the effective (i.e., multiplied by the photon-to-charge branching ratio) charge-carrier mobility as:

$$\phi\mu = -\epsilon_0 c(n_q + n_v)\frac{A_{eff}hc}{eE\lambda(1 - R_{pump} - T_{pump})}\left(\frac{\Delta T}{T}\right) \quad \text{(S4)}$$

We note that the sheet photoconductivity signal measured by OPTP arises from the contributions of both photogenerated free electrons and holes. Therefore, the extracted charge-carrier mobility is the electron-hole sum mobility.

To better capture the effect of charge carrier localization process on charge-carrier mobilities in silver-bismuth halides, Wright and Buizza developed a two-level mobility model.[1, 6] In this model, photoconductivity of the material is described as the sum of photoconductivities for two different states: a delocalized state and a localised state with population and mobility (n_{del}, μ_{del}) and (n_{loc}, μ_{loc}), respectively. Assuming a predominantly monomolecular recombination from the localised state, the carrier population is defined by the following set of coupled rate equations:

$$\begin{cases} \frac{dn_{del}}{dt} = -k_{loc} n_{del}(t) \\ \frac{dn_{loc}}{dt} = k_{loc} n_{del}(t) - k_1 n_{loc}(t) \end{cases} \quad \text{(S5)}$$

Here, k_{loc} and k_1 are the localization and monomolecular recombination rates, respectively. As described in details in Refs. 6 and 1, the resulting $\Delta T/T$ signal can be described as:

$$\frac{\Delta T}{T} = -\frac{Ne}{\epsilon_0 c(n_1 + n_3)A_{eff}} \left(\left(\mu_{del} - \frac{\mu_{loc} k_{loc}}{k_{loc} - k_1} \right) e^{-k_{loc} t} + \frac{\mu_{loc} k_{loc}}{k_{loc} - k_1} e^{-k_1 t} \right) \quad \text{(S6)}$$

Furthermore, to fit the experimental data reported in Figure 2a and Figure S5, we convoluted Equation S6 with a Gaussian function with broadening $\sigma = 250\, fs$ to account for the instrumental response function, as described in Reference 1.

Photoconductivity Spectra

Frequency-dependent photoconductivity spectra can give insight into the transport mechanism of free charge carriers in a semiconductor. Several different models (e.g., Drude, Drude-Lorentz, Drude-Anderson, Cole-Davison, Cole-Cole)[9] have been proposed to describe the photoconductivity spectra at THz frequencies and separate possible contributing scattering mechanisms.[10] Most of the proposed models are based on the Drude model of charge-carrier transport. This model describes the motion of charge carriers under an oscillating electric field with frequency ω, and assumes completely randomized charge-carrier velocity upon random scattering events with a characteristic scattering time τ.[11] The resulting Drude complex conductivity $\sigma_D(\omega)$ is given by:

$$\sigma_D(\omega) = \frac{ne^2\tau}{m^*}\left(\frac{1}{1 - i\omega\tau}\right) \tag{S7}$$

where m^* is the charge-carrier effective mass and n is the charge-carrier density. To better describe the photoconductivity response of semiconductor nanomaterials, the Drude-Smith model has been introduced as a phenomenological adaptation of the Drude model, which includes directional scattering events (i.e., backscattering) to describe scattering from surfaces.[12] Recently, Marcus and coworkers shown that this phenomenological model can be interpreted as a special case within the formalism of memory functions.[13] Here, the backscattering event is described by the phenomenological c coefficient (varying from 0 to -1), where $c = -1$ indicates a complete velocity reversal on the first collision. According to the Drude-Smith formulation, the complex conductivity can be expressed as:

$$\sigma_{DS}(\omega) = \frac{ne^2\tau}{m^*}\left(\frac{1}{1-i\omega\tau} + \frac{c}{(1-i\omega\tau)^2}\right). \quad \text{(S8)}$$

Furthermore, in principle, the Drude-Smith model would allow the extraction of charge-carrier mobility value defined as $\mu_{DS} = (1+c)\frac{e\tau}{m^*}$. However, we also note that in the limit of $c = -1$ μ_{DS} tends to infinity.[14] Therefore, in cases of substantial backscattering, μ_{DS} would be largely overestimated and its determination is avoided here.

Supporting Note 3: First-Principles Calculations of the Electronic Structure

Density functional theory (DFT) calculations were performed using the Vienna ab-initio simulation package (VASP) code[15] and the projector-augmented wave (PAW) method to describe core-valence electrons interactions.[16] We employed code-supplied PAW potentials that include semicore electrons explicitly, since they have been show to influence the electronic structure.[17] The valence configurations are reported in Table S1.We applied the Perdew-Burke-Ernzerhof (PBE)[18] form of the generalized gradient approximation to describe exchange-correlations interactions and the Tkatchenko-Scheffler scheme[19] to describe dispersive interactions. We set the plane-wave cutoff to 800 eV, used a 3×3×3 Γ-centered *k*-grid, and a total-energy threshold of 10^{-6} eV.

Table S1: Valence electron configurations of the PAW potentials used in the DFT calculations in VASP.

Element	Valence configuration
**Cs**	$5s^2 5p^6 6s^1$
**Ag**	$4s^2 4p^6 4d^{10} 5s^1$
**Bi**	$5s^2 5p^6 5d^{10} 6s^2 6p^3$
**Sb**	$4s^2 4p^6 4d^{10} 5s^2 5p^3$

**Br**	$3s^23p^63d^{10}4s^24p^5$

This setup was used to relax the cell shape and ionic positions of a series of 10 primitive cells with different volumes, until the maximum residual force was lower than 10^{-4} eV/Å. The lattice parameters were then obtained by fitting the calculated energy as a function of cell volume to a Birch-Murnaghn equation of state [20-21]. The resulting lattice parameters are in good agreement with the our experimental results and with those reported in the literature, with an error below 1% (see Table S2). The final structures used in the subsequent electronic band structure and effective mass calculations were obtained by relaxing the ionic degrees of freedom of the cell with optimized lattice parameters.

Table S2: DFT-calculated lattice constants (in Å) of $Cs_2AgBiBr_6$ ad $Cs_2AgSbBr_6$. The relative errors of the calculated parameter with respect to our measure and literature-reported values are shown in parenthesis.

Compound	DFT	Experimental	Literature
**$Cs_2AgBiBr_6$**	11.196	11.260 (0.6%)	11.271 (0.7%) Ref. 5
**$Cs_2AgSbBr_6$**	11.091	11.190 (0.9%)	11.158 (0.6%) Ref. 4

Spin-orbit coupling (SOC) was included in non-self-consistent calculations of the band structure from the charge densities calculated by self-consistent DFT. Including SOC has a strong impact ont the band gap and band dispersion at the CBM of $Cs_2AgBiBr_6$, while the effect is weaker for $Cs_2AgSbBr_6$ but not negligible. Specifically, SOC interactions reduce the fundamental indirect (X→L) gap of $Cs_2AgBiBr_6$ by 185 meV, whereas the reduction for $Cs_2AgSbBr_6$ is almost negligible (28 meV). Furthermore, including SOC changes the position of the direct gap in the Brillouin zone for both compounds from the $L = \left(\frac{1}{2}, \frac{1}{2}, \frac{1}{2}\right)$ to the $X = \left(\frac{1}{2}, 0, \frac{1}{2}\right)$ point. Moreover, SOC reduces the direct gap of $Cs_2AgBiBr_6$ by 721 meV and that of

$Cs_2AgSbBr_6$ by 150 meV. The stronger influence of SOC on the direct and indirect gaps of $Cs_2AgBiBr_6$ are caused by the strong spin-orbit interactions of Bi 6p orbitals participating in the CBM of the heavier Bi ion.

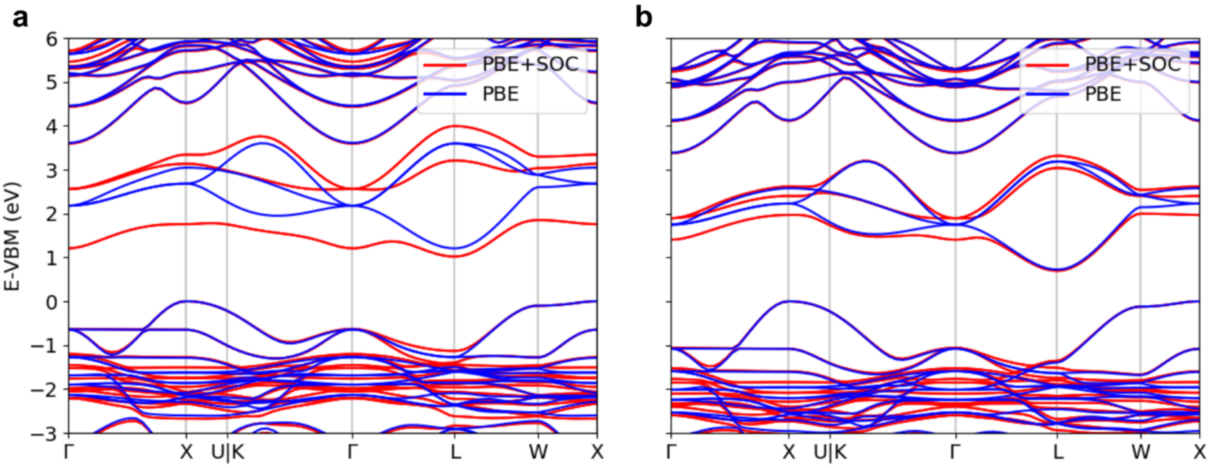

Figure S7 | Effect of spin-orbit coupling on the band structures of $Cs_2AgSbBr_6$ and $Cs_2AgBiBr_6$. (**a**) band structure of $Cs_2AgBiBr_6$ and (**b**) $Cs_2AgSbBr_6$, with (red lines) and without (blue lines) including spin-orbit coupling effects.

We calculated the effective mass tensors at the PBE+SOC level of theory by calculating the second-order partial derivatives using the valence and conduction band energy, $E_n(\boldsymbol{k})$, with respect to the $\boldsymbol{k}$ vector along the three Cartesian directions:

$$\left(\frac{1}{m^*}\right)ij = \frac{1}{\hbar^2}\frac{\partial^2 E_n(\boldsymbol{k})^2}{\partial k_i k_j} = \frac{1}{\hbar^2}\begin{pmatrix} \frac{\partial^2 E}{\partial k_x^2} & \frac{\partial^2 E}{\partial k_x \partial k_y} & \frac{\partial^2 E}{\partial k_x \partial k_z} \\ . & \frac{\partial^2 E}{\partial k_y^2} & \frac{\partial^2 E}{\partial k_y \partial k_z} \\ . & . & \frac{\partial^2 E}{\partial k_z^2} \end{pmatrix} \quad \text{(S9)}$$

The second derivatives were calculated numerically using second-order central differences on a 5 stencil grid with a converged step size of 0.01 $Bohr^{-1}$, as implemented in the EMC code.[22] After diagonalizing the effective mass tensor, we obtained the longitudinal ($m_{\parallel}$) and transversal ($m_{\perp}$) components of the electron and hole effective masses (see Table S1). For the electrons,

$m_{\|}$ corresponds to the path from L to Γ in the Brillouin zone (heavy electrons), while for holes, $m_{\|}$ corresponds to the direction from X to Γ (light holes).

Table S3: Effective mass tensor eigenvalues of $Cs_2AgBiBr_6$ and $Cs_2AgSbBr_6$ calculated around the VBM (X point) and CBM (L point). The masses are reported in the units of the electron rest mass m_0.

	**Electron Effective mass (m_0)**			**Hole Effective mass (m_0)**		
	$m_{\|,e}$	$m_{\perp,e}$	$m_{\perp,e}$	$m_{\|,h}$	$m_{\perp,h}$	$m_{\perp,h}$
**$Cs_2AgBiBr_6$**	0.44	0.29	0.29	0.17	0.68	0.68
**$Cs_2AgSbBr_6$**	0.30	0.26	0.26	0.15	0.73	0.73

Following a Drude model, the conductivity effective masses (see Table 1 in the main text) can be calculated as a harmonic mean of the longitudinal and transversal effective masses as $m^*_{cond} = 3\left(\frac{1}{m_{\|}} + \frac{2}{m_{\perp}}\right)^{-1}$. Furthermore, the effective mass anisotropy, λ, can be estimated as proposed by Schindlmayr[23]: $\lambda = \left(\frac{m_{\perp}}{m_{\|}}\right)^{1/3}$. The effective mass is isotropic when $\lambda \rightarrow 1$ and more anisotropic when λ is larger or smaller than one. For clarity, we quantify the degree of anisotropy by $\beta = |1 - \lambda|$, as shown in Table 1 in the Main Text. The hole effective masses are more anisotropic than electron effective masses ($\beta_h > 4\beta_e$) for both $Cs_2AgBiBr_6$ and $Cs_2AgSbBr_6$. Substituting Bi by Sb reduces the longitudinal electron effective mass and makes m_e^* more isotropic, while m_h^* becomes more anisotropic due to an increased transversal hole effective mass.

Biega et al.[24-25] reported that effective mass anisotropy is related to the deviation from the hydrogenic exciton model and estimated a larger exciton binding energy for $Cs_2AgSbBr_6$, compared with $Cs_2AgBiBr_6$.

Supporting Note 4: Discussion on free charge carriers and excitons populations

The analysis of the equilibrium exciton and free charge-carrier populations in semiconductors is conventionally based on Saha equation.[26-27] This formalism describes exciton formation and dissociation in terms of a chemical equilibrium between exciton (X) and free charge carriers, as $X \leftrightarrow e^- + h^+$. Here, the fraction of free charge-carrier n_f to the total excitation density n_{tot} is defined as branching ratio $\alpha = n_f / n_{tot}$, with $n_{tot} = n_f + n_x$ where n_x is the exciton density. According to the Saha formalism, the branching ratio is given by:

$$\frac{\alpha^2}{1-\alpha} = \frac{1}{n_{\mathrm{tot}}\lambda_{\mathrm{T}}^d} \exp\left(\frac{-E_{\mathrm{b}}}{k_{\mathrm{B}}T}\right) \tag{S10}$$

Where, we define the exciton De Broglie wavelength as $\lambda_T = h/\sqrt{2\pi\mu k_B T}$, and the exciton reduced mass as $\mu = m_e m_h/(m_e + m_h)$.

Here, we note that the Saha equation, given a few parameters such as the exciton binding energies, temperature, and the effective masses, estimates the free-carrier and exciton density at the thermal equilibrium. This equilibrium condition is not compatible with the transient experimental condition in OPTP measurements. Therefore, the α branching ratio obtained from the Saha equation does not directly reflect the photon-to-charge carrier branching ratio ϕ which factors the OPTP signal. Several processes and factors, such as exciton formation, excess excitation energy, and charge-carrier cooling could occur in the first few ps, thereby making it difficult to accurately predict ϕ.

While Saha equation predictions cannot be used to quantify ϕ, we note that they qualitatively predict a reduced free charge-carrier density in $Cs_2AgSbBr_6$ with respect to $Cs_2AgBiBr_6$. In the

hypothesis of similar exciton formation dynamics and charge-carrier cooling processes, this qualitatively supports our conclusion that increased excitonic effects could play a role in the observed lower effective mobilities for Sb-containing double perovskites.

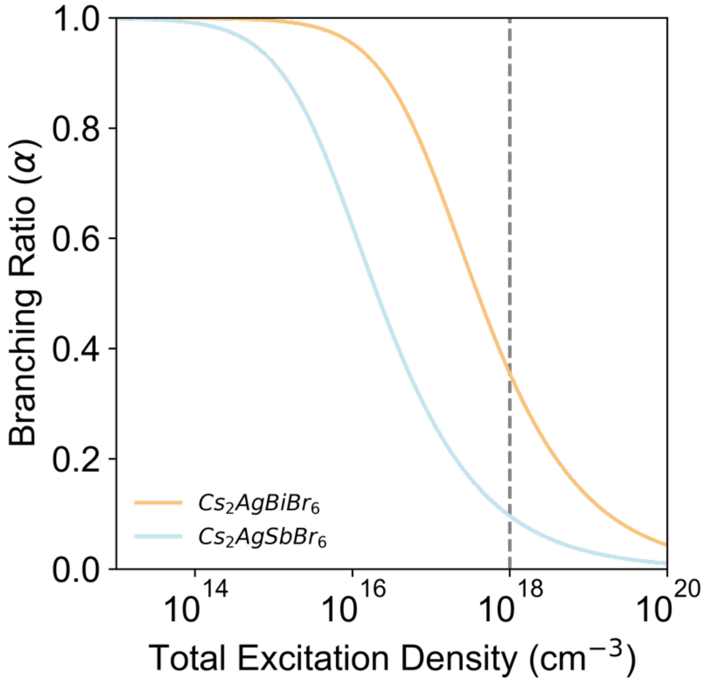

Figure S8 | Free charge carriers branching ratio for $Cs_2AgSbBr_6$ and $Cs_2AgBiBr_6$. Free-charge-carrier-to-total-carriers branching ratio plotted as a function of the total excitation density for $Cs_2AgSbBr_6$ (orange line) and $Cs_2AgBiBr_6$ (blue line). The branching ratio α is defined as $\alpha = n_f/n_{tot}$. Therefore, α =1 indicates the absence of stable excitons, while α =0 indicates the absence of free charge carriers. Branching ratio curves are obtained by solving the Saha equation at room temperature (298 K) with exciton binding energies and exciton-reduced mass estimated by Biega et al.[25]. The dashed line indicates a typical excitation density reached in OPTP experiments (i.e., 10^{18} cm^{-3})

Supporting References

(1) Buizza, L. R. V.; Wright, A. D.; Longo, G.; Sansom, H. C.; Xia, C. Q.; Rosseinsky, M. J.; Johnston, M. B.; Snaith, H. J.; Herz, L. M. Charge-Carrier Mobility and Localization in Semiconducting Cu_2AgBiI_6 for Photovoltaic Applications. *ACS En. Lett.* **2021,** *6,* 1729-1739.
(2) Seifert, T.; Jaiswal, S.; Martens, U.; Hannegan, J.; Braun, L.; Maldonado, P.; Freimuth, F.; Kronenberg, A.; Henrizi, J.; Radu, I.; Beaurepaire, E.; Mokrousov, Y.; Oppeneer, P. M.; Jourdan, M.; Jakob, G.; Turchinovich, D.; Hayden, L. M.; Wolf, M.; Münzenberg, M.; Kläui, M.; Kampfrath, T. Efficient metallic spintronic emitters of ultrabroadband terahertz radiation. *Nat. Photon.* **2016,** *10,* 483-488.
(3) Li, Z.; Kavanagh, S. R.; Napari, M.; Palgrave, R. G.; Abdi-Jalebi, M.; Andaji-Garmaroudi, Z.; Davies, D. W.; Laitinen, M.; Julin, J.; Isaacs, M. A.; Friend, R. H.; Scanlon, D. O.; Walsh, A.; Hoye, R. L. Z. Bandgap lowering in mixed alloys of $Cs_2Ag(Sb_xBi_{1-x})Br_6$ double perovskite thin films. *J. Mater. Chem. A* **2020,** *8,* 21780-21788.
(4) Wei, F.; Deng, Z.; Sun, S.; Hartono, N. T. P.; Seng, H. L.; Buonassisi, T.; Bristowe, P. D.; Cheetham, A. K. Enhanced visible light absorption for lead-free double perovskite $Cs_2AgSbBr_6$. *Chem. Commun.* **2019,** *55,* 3721-3724.
(5) McClure, E. T.; Ball, M. R.; Windl, W.; Woodward, P. M. Cs_2AgBiX_6 (X = Br, Cl): New Visible Light Absorbing, Lead-Free Halide Perovskite Semiconductors. *Chem. Mater.* **2016,** *28,* 1348-1354.
(6) Wright, A. D.; Buizza, L. R. V.; Savill, K. J.; Longo, G.; Snaith, H. J.; Johnston, M. B.; Herz, L. M. Ultrafast Excited-State Localization in $Cs_2AgBiBr_6$ Double Perovskite. *J. Phys. Chem. Lett.* **2021,** *12,* 3352-3360.
(7) Wehrenfennig, C.; Eperon, G. E.; Johnston, M. B.; Snaith, H. J.; Herz, L. M. High Charge Carrier Mobilities and Lifetimes in Organolead Trihalide Perovskites. *Adv. Mater.* **2014,** *26,* 1584-1589.
(8) Joyce, H. J.; Boland, J. L.; Davies, C. L.; Baig, S. A.; Johnston, M. B. J. S. S.; Technology A review of the electrical properties of semiconductor nanowires: insights gained from terahertz conductivity spectroscopy. **2016,** *31,* 103003.
(9) Joyce, H. J.; Boland, J. L.; Davies, C. L.; Baig, S. A.; Johnston, M. B. A review of the electrical properties of semiconductor nanowires: insights gained from terahertz conductivity spectroscopy. *Semicond. Sci. Technol.* **2016,** *31,* 103003.
(10) Ulatowski, A. M.; Herz, L. M.; Johnston, M. B. Terahertz Conductivity Analysis for Highly Doped Thin-Film Semiconductors. *J. Infrared Millimeter Waves* **2020,** *41,* 1431-1449.
(11) Herz, L. M. Charge-Carrier Mobilities in Metal Halide Perovskites: Fundamental Mechanisms and Limits. *ACS En. Lett.* **2017,** *2,* 1539-1548.
(12) La-o-vorakiat, C.; Salim, T.; Kadro, J.; Khuc, M.-T.; Haselsberger, R.; Cheng, L.; Xia, H.; Gurzadyan, G. G.; Su, H.; Lam, Y. M.; Marcus, R. A.; Michel-Beyerle, M.-E.; Chia, E. E. M. Elucidating the role of disorder and free-carrier recombination kinetics in $CH_3NH_3PbI_3$ perovskite films. *Nat. Commun.* **2015,** *6,* 7903.
(13) Chen, W.-C.; Marcus, R. A. The Drude-Smith Equation and Related Equations for the Frequency-Dependent Electrical Conductivity of Materials: Insight from a Memory Function Formalism. *ChemPhysChem* **2021,** *22,* 1667-1674.
(14) Motti, S. G.; Krieg, F.; Ramadan, A. J.; Patel, J. B.; Snaith, H. J.; Kovalenko, M. V.; Johnston, M. B.; Herz, L. M. CsPbBr3 Nanocrystal Films: Deviations from Bulk Vibrational and Optoelectronic Properties. *Adv. Funct. Mater.* **2020,** *30,* 1909904.
(15) Kresse, G.; Furthmüller, J. Efficient iterative schemes for ab initio total-energy calculations using a plane-wave basis set. *Phys. Rev. B* **1996,** *54,* 11169-11186.
(16) Kresse, G.; Joubert, D. From ultrasoft pseudopotentials to the projector augmented-wave method. *Phys. Rev. B* **1999,** *59,* 1758-1775.
(17) Leppert, L.; Rangel, T.; Neaton, J. B. Towards predictive band gaps for halide perovskites: Lessons from one-shot and eigenvalue self-consistent *GW*. *Phys. Rev. Mater.* **2019,** *3,* 103803.

(18) Perdew, J. P.; Burke, K.; Ernzerhof, M. Generalized Gradient Approximation Made Simple. *Phys. Rev. Lett.* **1996,** *77,* 3865-3868.
(19) Tkatchenko, A.; Scheffler, M. Accurate Molecular Van Der Waals Interactions from Ground-State Electron Density and Free-Atom Reference Data. *Phys. Rev. Lett.* **2009,** *102,* 073005.
(20) Birch, F. Finite Elastic Strain of Cubic Crystals. *Phys. Rev.* **1947,** *71,* 809-824.
(21) Murnaghan, F. D. The Compressibility of Media under Extreme Pressures. *PNAS* **1944,** *30,* 244-247.
(22) Fonari, A.; Sutton, C. Effective Mass Calculator. **2012**.
(23) Arno, S. Excitons with anisotropic effective mass. *Eur. J. Phys.* **1997,** *18,* 374.
(24) Biega, R.-I.; Chen, Y.; Filip, M. R.; Leppert, L. Chemical Mapping of Excitons in Halide Double Perovskites. *Nano Letters* **2023**, 10.1021/acs.nanolett.3c02285.
(25) Biega, R.-I.; Filip, M. R.; Leppert, L.; Neaton, J. B. Chemically Localized Resonant Excitons in Silver–Pnictogen Halide Double Perovskites. *J. Phys. Chem. Lett.* **2021,** *12,* 2057-2063.
(26) Saha, M. N. On a Physical Theory of Stellar Spectra. *Proceedings of the Royal Society of London. Series A, Containing Papers of a Mathematical and Physical Character* **1921,** *99,* 135-153.
(27) Righetto, M.; Giovanni, D.; Lim, S. S.; Sum, T. C. The photophysics of Ruddlesden-Popper perovskites: A tale of energy, charges, and spins. *App. Phys. Rev.* **2021,** *8,* 011318.
